# Research on the Force-Sensitive Characteristic of InAs QD Embedded in HEMT

**DOI:** 10.3390/mi12111413

**Published:** 2021-11-18

**Authors:** Rui-Rong Wang, Hao Guo, Jun Tang, Jin-Ping Liu, Li-Shuang Liu

**Affiliations:** 1 Key Laboratory of Instrumentation Science and Dynamic Measurement, Shanxi Province Key Laboratory of Quantum Sensing and Precision Measurement, School of Instrument and Electronics, North University of China, Taiyuan 030051, China; wangruirong@yeah.net (R.-R.W.); guohaonuc@163.com (H.G.); tangjun@nuc.edu.cn (J.T.); s1906033@163.com (J.-P.L.); 2 Department of Electronic Engineering, Taiyuan Institute of Technology, Taiyuan 030008, China

**Keywords:** InAs QD, HEMT, force-sensitive

## Abstract

A force-sensitive structure of an InAs Quantum Dot (QD) embedded in a high electron mobility transistor (HEMT) is presented in this paper. The size of an InAs QD is about 30 nm prepared by the S-K growth mode, and the force-sensitive structure is fabricated by molecular beam epitaxy (MBE). The force-sensitivity characteristic of the QD HEMT is studied by the electrical and mechanical properties. The electrical characteristics show that the InAs QD-HEMT has linear, cut-off, and saturation operating states, and produces different output currents under different gate voltages, which shows that the structure is reasonable. Furthermore, the results of the output characteristics under different pressure show that the output voltage of the QD-HEMT decreases with the increase in pressure, which indicates that the InAs QD-HEMT has a vital mechanical–electrical coupling characteristic. The output voltage of the InAs QD-HEMT in the range of 0–100 kPa shows that the sensitivity was 1.09 mV/kPa.

## 1. Introduction

The Micro-ElectroMechanical System (MEMS) has a broad application in aerospace, space communication, satellite, military, and nuclear fields [1,2,3] based on the advantages of being small in size, lightweight, with a fast response, low-power consumption, easy to miniature and integrate [4,5,6,7,8]. The demand for the sensitivity of MEMS sensors is also constantly improved by the demand for high precision in these fields [9].

The conventional pressure sensors are mainly based on the piezoresistive, capacitive, and piezoelectric methods. Among them, the piezoelectric pressure sensor has low sensitivity. Using a high electron mobility transistor (HEMT) as the sensing element can improve sensitivity [10,11]. Tan, X. et al. [12]. proposed a wheat-stone bridge pressure sensor based on the AlGaN/GaN HEMT, which realizes the detection of mechanical signals, but with a low sensitivity of 1.25 μV/kPa/V. Dzuba, J. et al. [13]. presented an AlGaN/GaN circular HEMT pressure sensing device with a detecting sensitivity up to 4.4 pc/kPa, realizing the detection of the mechanical signal by measuring the change of charges. Chen, X. et al. [14]. proposed the method of embedded technology to achieve a couple of HEMT and MEMS pressure sensors.

The QD embedded in the HEMT force-sensitive sensor has been applied to the MEMS sensor due to the advantages of high mobility, high sensitivity, high bandwidth, and excellent other electrical properties [15,16]. In this paper, a force-sensitive structure of the InAs QD embedded in the HEMT is presented for high-sensitivity detection. The InAs QD is prepared by the S-K growth mode, and the force-sensitive structure is fabricated by molecular beam epitaxy (MBE). The electrical and mechanical characteristics of the structure are tested. Additionally, the results show that the structure of the InAs QD-HEMT has a strong mechanical-electrical coupling characteristic. The sensitivity of the InAs QD-HEMT structure was 1.09 mV/kPa in the range of 0–100 kPa.

## 2. Structure Design

**Figure 1:**
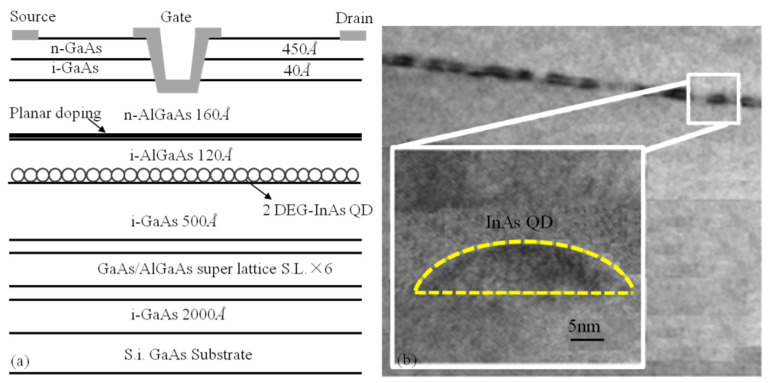
(**a**) The schematic of InAs QD-HEMT structure (**b**) The cross-section TEM of QD-HEMT.

In this paper, a 2-DEG (two-dimensional electron gas) InAs QD embedded in an HEMT force-sensitive structure was designed, which was fabricated using the MBE technique. Firstly, a 200-nanometer high-purity GaAs buffer layer was grown on a semi-insulating GaAs substrate, avoiding the influence of substrate defects, harmful impurities, and thermal conversion on the active layer. Without a buffer layer, the mobility of the active layer showed an apparent decline toward the substrate. Meanwhile, the buffer layer could also smooth the surface by the roughness reduction on the top surface. Then, a high concentration GaAs/AlGaAs superlattice layer was grown and integrated with the transition region, and the high-purity GaAs channel layer was formed to improve the characteristics of transconductance and breakdown simultaneously. After, the growth of the InAs quantum dot layer was prepared. A high-purity AlGaAs with a thickness of 12 nm was grown as the isolation layer to overcome the disadvantage of the low Schottky barrier in the Si plane doped isolation layer. Then, the n-AlGaAs barrier layer with a thickness of 16 nm was grown as the electron supply layer for Schottky contact on this layer. After that, another high-purity GaAs isolation layer was deposited on the n-AlGaAs barrier layer. Finally, a high-doped GaAs ohmic contact layer with a thickness of 45 nm was used to achieve the source and drain ohmic contacts. The gate length, gate width, and channel thickness of the InAs QD HEMT force-sensitive structures are 0.5 µm, 176 µm, and 50 nm, respectively. The schematic of the InAs QD-HEMT structure is shown in Figure 1, and the size of the InAs QD embedded in the HEMT structure is about 30 nm.

## 3. Experimental Test

### 3.1. Electrical Characteristic

**Figure 2:**
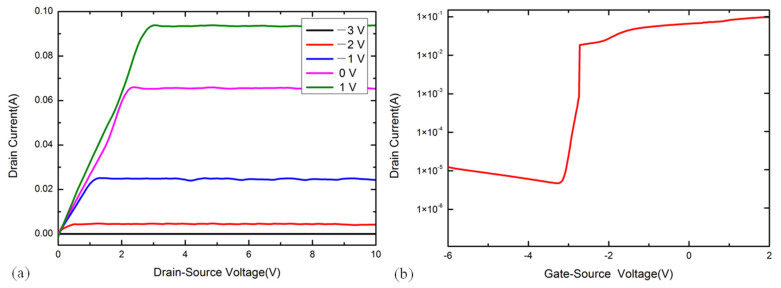
The electrical characteristic of InAs QD-HEMT (**a**) The output characteristic (I_DS_-V_DS_) (**b**) The transfer characteristic.

The electrical performance of the InAs QD-HEMT force-sensitive structure was tested using a Keithley 4200 semiconductor characteristic analyzer at room temperature, and the corresponding results are shown in Figure 2. Figure 2a shows the output characteristic curves of the InAs QD-HEMT under the gate voltage of V_GS_ = −3, −2, −1, 0, and 1 V in the V_DS_ range of 0–10 V. Figure 2b shows the transfer characteristic curve at the gate voltage of V_DS_ = 5 V. The output characteristic curve and transfer characteristic curve of the InAs QD-HEMT are relatively smooth. The result of output characteristic shows that the InAs QD-HEMT has linear, cut-off, and saturation operating states, and produces different output currents under different gate voltages, indicating that the design of the InAs QD-HEMT force-sensitive structure is reasonable.

### 3.2. The Detection Principle of Force-Sensitive

**Figure 3:**
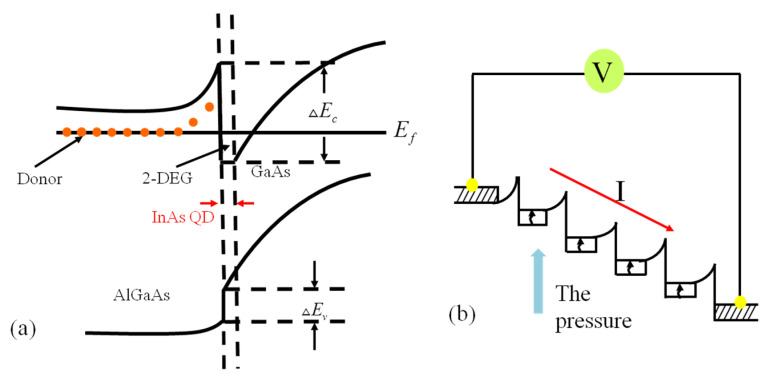
(**a**) The energy level diagram of QD-HEMT (**b**) the change of current flow under pressure.

The energy level diagram of the QD-HEMT is shown in Figure 3a. The QD-HEMT is used as the force-sensitive unit of the MEMS sensor. When the force is applied to the sensor, the energy band structure and internal lattice of the QD-HEMT force-sensitive unit will be changed due to the force [17].The deformation of the channel layer will cause the change of free electron mobility; the change of internal lattice leads to lattice expansion, which results in the enhancement of the scattering effect and affects the free electron mobility further; the change of the energy band structure will affect the electron transfer from the valence band to the conduction band, and the free electron concentration, resulting in the change of conductivity and mobility. These factors will affect the 2-DEG in the QD-HEMT structure, resulting in the change of output current, which is shown as the change of the QD-HEMT output current (I_DS_) macroscopically. Through this physical process, the transformation is realized from a mechanical signal to an electrical signal. The change of current flow under pressure is shown in Figure 3b.

### 3.3. Force-Sensitive Characteristic

**Figure 4:**
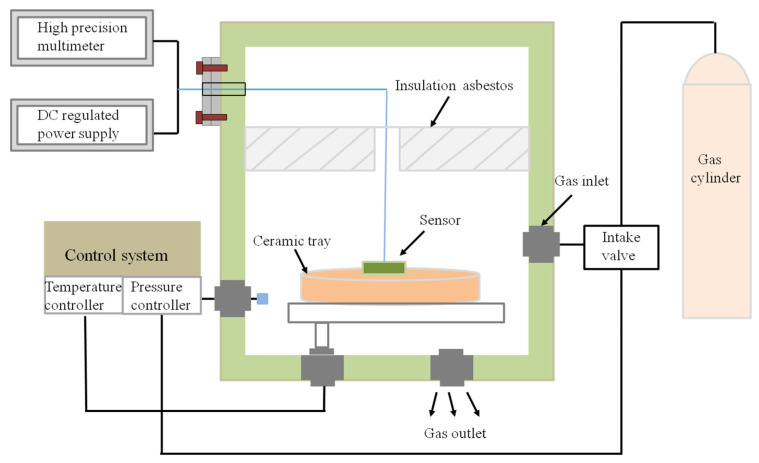
The force-sensitive measurement system.

In order to study the mechanical characteristics of the InAs QD-HEMT sensitive structure, the device was tested under a pressure of 0–100 kPa at room temperature by using the JT-1500 high-temperature and pressure composite testing platform (Chengdu Jiangtai Co., Ltd. Sichuan, China), and the test system is shown in Figure 4. The composite test platform was composed of the control system, the sealed high-temperature pressure tank, and an argon cylinder. In this platform, the reference temperature and pressure sensors were used, respectively, to feedback the temperature and pressure; thereby, these two parameters could be adjusted by the control system.

**Figure 5:**
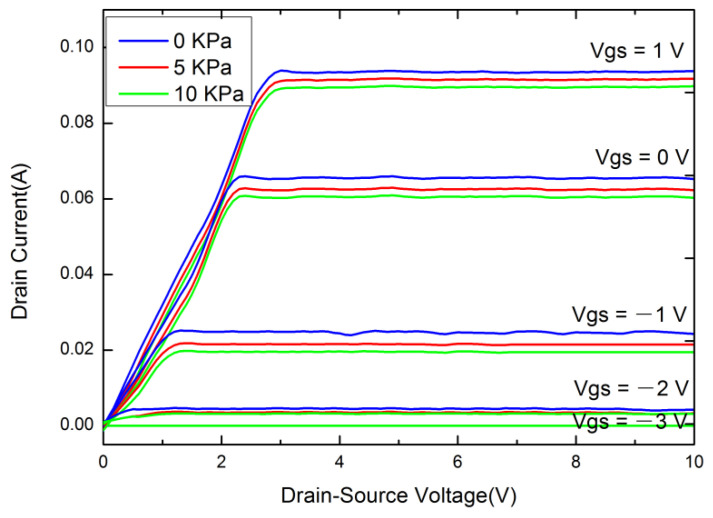
The output characteristic of QD-HEMT under the stress of 5 and 10 KPa.

The output characteristics of the QD-HEMT were tested under the stress of 5 and 10 KPa, and the results are shown in Figure 5. The output current of the QD-HEMT changes obviously under the stress of 5 and 10 KPa, indicating that the internal transport mechanism of the sensitive unit has changed; that is, the drift of carriers is affected under the action of stress due to the change of energy level structure, so as to change the size of the space charge region and change the channel width of the carriers. Finally, the output current is changed by realizing the electromechanical conversion from a mechanical signal to an electrical signal, manifesting that the QD-HEMT has a strong electromechanical coupling characteristic.

**Figure 6:**
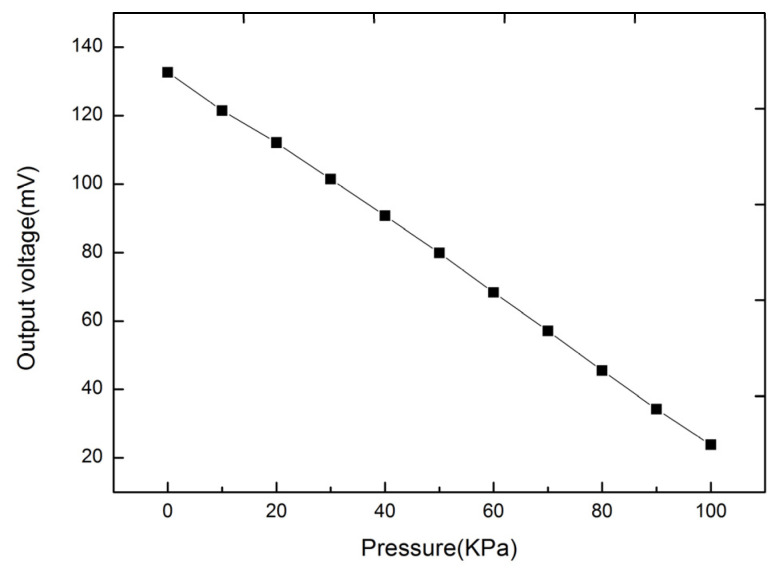
The force-sensitive characteristic of InAs QD-HEMT.

The sensitivity of the InAs QD-HEMT structure was tested in the stress range of 0–100 KPa. Figure 6 shows the output voltage V_DS_ as the function of the stress at the V_gs_ = 1 V. According to the test results, the sensitivity of the InAs QD-HEMT is 1.09 mV/KPa.

## 4. Conclusions

In this paper, a force-sensitive structure based on the InAs QD embedded in the HEMT was proposed. It was shown that the designed structure is reasonable through the electrical characteristic test of the force-sensitive structure. The mechanical characteristic test shows that the structure has strong mechanical and electrical coupling characteristics. In the range of 0–100 KPa, the sensitivity of the InAs QD-HEMT structure is 1.09 mV/KPa, which realizes the high-sensitive detection of mechanical signals.
